# Novel Evoked Synaptic Activity Potentials (ESAPs) Elicited by Spinal Cord Stimulation

**DOI:** 10.1523/ENEURO.0429-22.2023

**Published:** 2023-05-16

**Authors:** Mahima Sharma, Vividha Bhaskar, Lillian Yang, Mohamad FallahRad, Nigel Gebodh, Tianhe Zhang, Rosana Esteller, John Martin, Marom Bikson

**Affiliations:** 1 Neural Engineering Laboratory, Department of Biomedical Engineering, The City College of the City University of New York, City College Center for Discovery and Innovation, New York, NY 10031; 2 Department of Molecular, Cellular and Biomedical Sciences, The City College of the City University of New York, City College Center for Discovery and Innovation, New York, NY 10031; 3 Boston Scientific Neuromodulation Research and Advanced Concepts, Valencia, CA 91355

**Keywords:** EMG, epidural recording, evoked compound action potential, evoked synaptic activity potentials

## Abstract

Spinal cord stimulation (SCS) evokes fast epidural evoked compound action potential (ECAP) that represent activity of dorsal column axons, but not necessarily a spinal circuit response. Using a multimodal approach, we identified and characterized a delayed and slower potential evoked by SCS that reflects synaptic activity within the spinal cord. Anesthetized female Sprague Dawley rats were implanted with an epidural SCS lead, epidural motor cortex stimulation electrodes, an epidural spinal cord recording lead, an intraspinal penetrating recording electrode array, and intramuscular electromyography (EMG) electrodes in the hindlimb and trunk. We stimulated the motor cortex or the epidural spinal cord and recorded epidural, intraspinal, and EMG responses. SCS pulses produced characteristic propagating ECAPs (composed of P1, N1, and P2 waves with latencies <2 ms) and an additional wave (“S1”) starting after the N2. We verified the S1-wave was not a stimulation artifact and was not a reflection of hindlimb/trunk EMG. The S1-wave has a distinct stimulation-intensity dose response and spatial profile compared with ECAPs. 6-Cyano-7-nitroquinoxaline-2,3-dione (CNQX; a selective competitive antagonist of AMPA receptors (AMPARs)] significantly diminished the S1-wave, but not ECAPs. Furthermore, cortical stimulation, which did not evoke ECAPs, produced epidurally detectable and CNQX-sensitive responses at the same spinal sites, confirming epidural recording of an evoked synaptic response. Finally, applying 50-Hz SCS resulted in dampening of S1-wave but not ECAPs. Therefore, we hypothesize that the S1-wave is synaptic in origin, and we term the S1-wave type responses: evoked synaptic activity potentials (ESAPs). The identification and characterization of epidurally recorded ESAPs from the dorsal horn may elucidate SCS mechanisms.

## Significance Statement

Spinal cord stimulation (SCS) is an established treatment for chronic pain and has applications to other disorders and neurorehabilitation. Notwithstanding decades of trials and research, questions remain about SCS mechanisms of action, and indicators thereof. Recent technological developments have enabled the detection of evoked compound action potential (ECAP), reflecting synchronous activity of the dorsal column axons activated by SCS. However, ECAP is not a direct measure of sensory processing in the dorsal horn. Here, we identify and characterize a novel electrophysiological signal that is evoked and detectable by epidural SCS electrodes and reflects spinal synaptic currents. This new signal, termed an evoked synaptic activity potential (ESAP), is thus a novel means with which to interrogate spinal gray matter circuits during SCS.

## Introduction

Epidural spinal cord stimulation (SCS) for neuropathic pain is thought to engage inhibitory dorsal horn mechanisms, whether via synaptic activation following antidromic stimulation of dorsal column fibers, consistent with Gate Control Theory (Shealy et al., 1970), or through more subtle, slower acting mechanisms (Titus et al., 2021). Dorsal column stimulation is sensed by evoked electrical responses representing the summed activity of dorsal column (Aβ) axons, whose synchronous activation produces a propagating evoked compound action potential (ECAP) characterized by P1, N1, and P2 waves, with latencies <2 ms proximal to the stimulation site (Cioni and Meglio, 1986; Parker et al., 2013; Cedeño et al., 2022; Dietz et al., 2022). The stimulation current threshold for ECAPs correlates with patient reported paresthesia and discomfort thresholds, and ECAP recording can be performed using contacts on the therapeutic stimulation leads, supporting the use of ECAPs in closed-loop SCS systems (Parker et al., 2012). However, dorsal column activity (i.e., the ECAP) is not itself directly indicative of pain or analgesia (Pilitsis et al., 2021; Vallejo et al., 2021), inviting a search for other signals that may better indicate pain relief by SCS.

Experimental electrophysiology involving the spinal cord, spanning decades, identified field potentials beyond the ECAP with slower time-courses ranging from a few milliseconds to tens or hundreds of milliseconds after a given stimulus (Wall, 1958; Cervero et al., 1978; Yates et al., 1982; Ondrejcák et al., 2005). Historically, studies of slow spinal potentials supported development of theories for pain manifestation and its control (Wall, 1958), including Gate Control Theory, and initial mechanistic hypotheses about SCS were tested through measurement of longer-latency “prolonged small fiber after discharge” (PSAD; Shealy et al., 1970). Slow spinal potentials may correlate with modulation of afferent input (e.g., primary afferent depolarization), interneuron activity, or dorsal horn excitability (Wall, 1958; Manjarrez et al., 2003; Contreras-Hernández et al., 2015, 2022) and may therefore provide more information about the spinal (pain) state than ECAPs alone. However, fundamental studies on slower potentials used electrodes not suitable for clinical applications. Analysis of epidurally recorded evoked potentials for SCS has focused on the fast ECAPs (Parker et al., 2012; Anaya et al., 2020); slower evoked responses have been sparsely noted but unreliably, at high clinical intensities, and topically described as signs of patient discomfort (Parker et al., 2012) or muscle activation (Falowski et al., 2022).

Here, we use a rodent model to identify and characterize an electrophysiological signal evoked and detectable by epidural SCS electrodes yet reflecting intraspinal synaptic activity, and with a threshold above ECAP thresholds but below electromyography (EMG) thresholds, and a latency after ECAP onset but before EMG onset. We show this S1-wave reflects gray-matter excitatory synaptic currents, as part of a type of responses that we term evoked synaptic activity potentials (ESAPs). The distinct etiology of ESAPs (gray matter synaptic processing) versus ECAPs (white matter conduction) is reflected in distinct (spatiotemporal, stimulation intensity, single pulse vs tonic) responses to SCS. Building on the work described here, future studies on whether and how ESAPs indicate effects of SCS and/or dorsal horn state (e.g., sensory/proprioceptive processing) are warranted.

## Materials and Methods

### Ethics statement

All experiments were performed in accordance with the NIH’s *Guide for the Care and Use of Laboratory Animals*. Care and treatment of the animals conformed to protocols approved by the Institutional Animal Care and Use Committee of the City College of the City University of New York.

### Animal subjects

A total of 47 adult female Sprague Dawley rats, weighing 250–300 g on the day of the experiment, were used for all procedures. Rats were housed in groups of two to three per filter-top polycarbonate cages at our climate-controlled vivarium with the standard 12/12 h light/dark cycle, room temperature of 22.5 ± 0.9°C, relative humidity of 36 ± 4%, food and water *ad libitum*. Rats had a minimum 3-d acclimation period before starting any experiment. All procedures were conducted between 10 A.M. and 5 P.M. local time.

### Anesthesia and animal preparation

Rats were anesthetized using intraperitoneal injection of a mixture of ketamine and xylazine (70 mg/kg; 6 mg/kg). Care was taken to maintain a relatively stable anesthetic state and body temperature. Body temperature was monitored with a rectal probe and maintained at 37 ± 1°C during the entire experimental procedure, using a feedback-controlled warming pad (Physio Suite, Kent Scientific). Ophthalmic ointment was applied to the eyes to prevent ocular dryness. The scalp, back and left hindlimb were shaved to facilitate craniotomy, laminectomy, and optimal measurements from EMG recording electrodes.

To assess the appropriate depth of anesthesia, periodic monitoring was conducted of respiratory and heart rates (Physio Suite, Kent Scientific), muscle tone, the absence of vibrissae whisking, and the absence of hindlimb withdrawal to foot pinch. The required anesthetic depth was maintained throughout the experiment with regular intraperitoneal administration of supplemental doses of ketamine (25–35 mg/kg) every 90 min (Borrell et al., 2017).

### Surgery

All the rats were subjected to laminectomy; 10 rats also underwent a craniotomy. During all surgical procedures, hemostasis was generally achieved through gentle pressure and the wounds were irrigated using sterile saline. Warm sterile saline was applied to surgical wounds to protect the tissues from desiccation during the entire experimental procedure.

#### Laminectomy

Rats were placed in a stereotaxic frame (David Kopf Instruments) after induction of anesthesia. Following shaving of the back and subcutaneous administration of 0.5% lidocaine (Henry Schein Medical) at the incision site, a midline incision was made using the scalpel blade over the skin of the back to expose the T10–L2 vertebrae. The overlying musculature and fascia were cleared. After identifying the T13 vertebrae, a bilateral laminectomy was performed at the T10 and T11 vertebrae, to expose T12–L1 spinal segments. A hemi-laminectomy was performed on the left side of L1 and bilateral laminectomy at the L2 vertebrae to expose the L4–S3 segments of the spinal cord. Muscles on each side of midline were expanded slowly using a self-retaining retractor placed under the muscle. To stabilize the spinal cord for neural recordings, the L3 lamina was clamped. An incision was made in the dura at L4/L5 spinal segments and the pia was microdissected for insertion of the intraspinal electrode array and/or intrathecal drug administration.

#### Craniotomy

After placing the rats in a stereotaxic frame (David Kopf Instruments) and adjusting the incisor bar to ensure a flat skull position (equal heights of λ and bregma skull points), a midline scalp incision was made. Craniotomy was performed over the hindlimb representation of the motor cortex (1.7 ± 0.4 mm posterior to bregma, 1.5 ± 0.5 mm lateral to midline) by thinning the skull over the primary motor cortex (5 × 5 mm) using a small burr. The thinned bone flap was then peeled off using fine micro-forceps. The general location of the craniotomy was guided by previous motor mapping studies of the rat (Fonoff et al., 2009; Frost et al., 2013) and was made intentionally larger than the anticipated location of the hindlimb motor cortex to account for slight variability among animals and ensure ample space for the electrode placement.

#### Placement of epidural spinal leads

**Figure 1.**
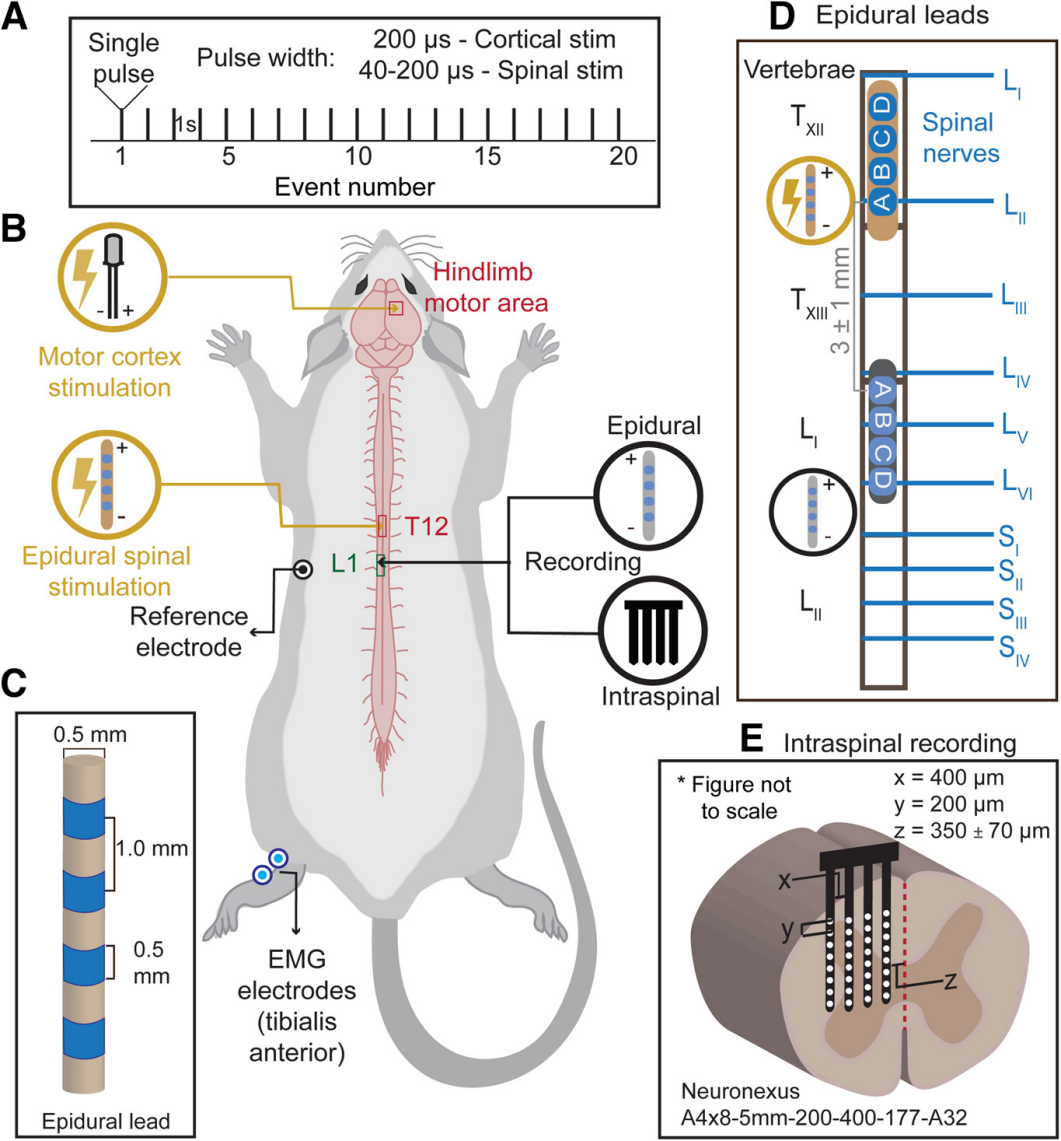
Experimental setup combining epidural cortical stimulation, epidural spinal stimulation, epidural spinal recording, invasive spinal recording, and EMG in rodent model. ***A***, Representative cortical and epidural spinal stimulation pulse trains of 20 low-frequency pulses, with pulse width of 200 μs per phase and/or 40–200 μs per phase, respectively. ***B***, Rat model depicting different electrodes sites. The right hindlimb representation area (HLA) of the motor cortex (M1) is stimulated using epidural bipolar stimulating electrode. Epidural leads are positioned on the midline of the spinal cord at T12–T13 segment and L1 vertebral segment to stimulate (orange) and record (gray) from the spinal cord, respectively. A four-shank silicon array (black) records the intraspinal responses. A reference electrode is placed under the skin. Percutaneous nickel-chrome wire electrodes (deinsulated 1 mm from the tip) bilaterally implanted into the tibialis anterior muscles record EMG responses. ***C***, Epidural lead with four electrodes of 0.5-mm length each, separated by 1 mm. The diameter of the lead is 0.5 mm. ***D***, Representative placement of the four-electrode epidural leads (contacts A, B, C, and D oriented as shown) along the spinal cord. Stimulating (orange) and recording (gray) epidural leads are placed at the T12 vertebral segment and L1 vertebral segment, respectively, with the interelectrode distance between electrode contact A of the epidural stimulating lead and electrode contact A of the epidural recording lead of 3 ± 1 mm. ***E***, Cross section of spinal cord showing the placement of electrode for intraspinal recording. A four-shank Neuronexus silicon array is inserted transversely into the left side of the spinal cord to a depth of 1.7 ± 0.2 mm from the pial surface. *Figure Contributions*: Mahima Sharma, Vividha Bhaskar, John Martin, Tianhe Zhang, Rosana Esteller, Lillian Yang, and Nigel Gebodh designed the figure.

We used two custom-made cylindrical four-electrode leads (0.5-mm diameter; 1-mm interelectrode distance; 0.5 mm wide; Boston Scientific Neuromodulation; Fig. 1*C*,*D*), one to stimulate the spinal cord and other to record the epidural spinal responses. Both leads were placed epidurally over the midline of dorsal spinal cord. The epidural stimulation lead was inserted in the caudal direction through the laminectomy site at T10–T11 vertebral levels. The stimulation lead was adjusted so that active contacts were at the T12/T13 vertebral levels analogous to previous studies (Shechter et al., 2013; Tao et al., 2021). Vertebral locations were chosen based on prior work demonstrating that vertebral levels T12–L1 correspond to lumbar spinal dermatomes (L2–L5) often explored in chronic pain and motor systems research (Gelderd and Chopin, 1977; Swett and Woolf, 1985; Gerasimenko et al., 2019). The epidural recording lead was inserted in the rostral direction through the laminectomy site at L1 vertebra. The recording lead was adjusted so that the contacts were at T13/L1 vertebral levels to record dorsal epidural spinal responses from spinal lumbar segments L2–L5 (Dietz et al., 2022).

### Electrophysiology

#### Cortical and epidural spinal stimulation

For stimulation of both the hindlimb representation area (HLA) and epidural dorsal columns, a constant current stimulator (A-M Systems Model 2100) was set to deliver constant current symmetric biphasic stimulation either through the M1 cortical electrode or the epidural spinal lead, respectively. The M1 bipolar cortical stimulating electrode (PlasticsOne) was placed epidurally over HLA. The electrode was placed in the center of the hindlimb field on the motor cortex (1.8 ± 0.4 mm posterior to bregma, 1.6 ± 0.3 mm lateral to midline; Barth et al., 1990; Frost et al., 2013).

The epidural spinal stimulation lead had four electrode contacts designated A, B, C, D; with A electrode nearest lead terminus. The interelectrode distance between the A electrodes of the epidural stimulating lead and the epidural recording lead was 3 ± 1 mm (Fig. 1*D*). Stimulation was applied using adjacent bipolar electrodes, delivering biphasic pulses with either leading anodic (rostral to caudal: − +) or cathodic (rostral to caudal: + −; unless otherwise indicated).

We optimized electrode placement to normalize the stimulation parameters used in this experimental series. At the start of experiments, brief trains of biphasic pulses (333 Hz, contact A-B) were applied, as previously described for M1 mapping experiments (Song and Martin, 2017; Takemi et al., 2017); current amplitude was incremented (up to 0.8 mA) until either contractions or movement of the left hindlimbs were visually observed. The spinal stimulation lead was repositioned caudally/rostrally in ∼1-mm increments, as necessary, to elicit an extension of one or more joints of the hindlimb. Next, low-frequency epidural SCS was delivered through the designated stimulation lead while recording from the other lead (Fig. 1*A*). The stimulation or recording leads were repositioned caudally/rostrally in ∼1-mm increments and/or different electrode pairs were tested, as necessary, to determine the best site for stimulation and recording of a delayed response (e.g., S1 wave).

#### Epidural recording

**Figure 2.**
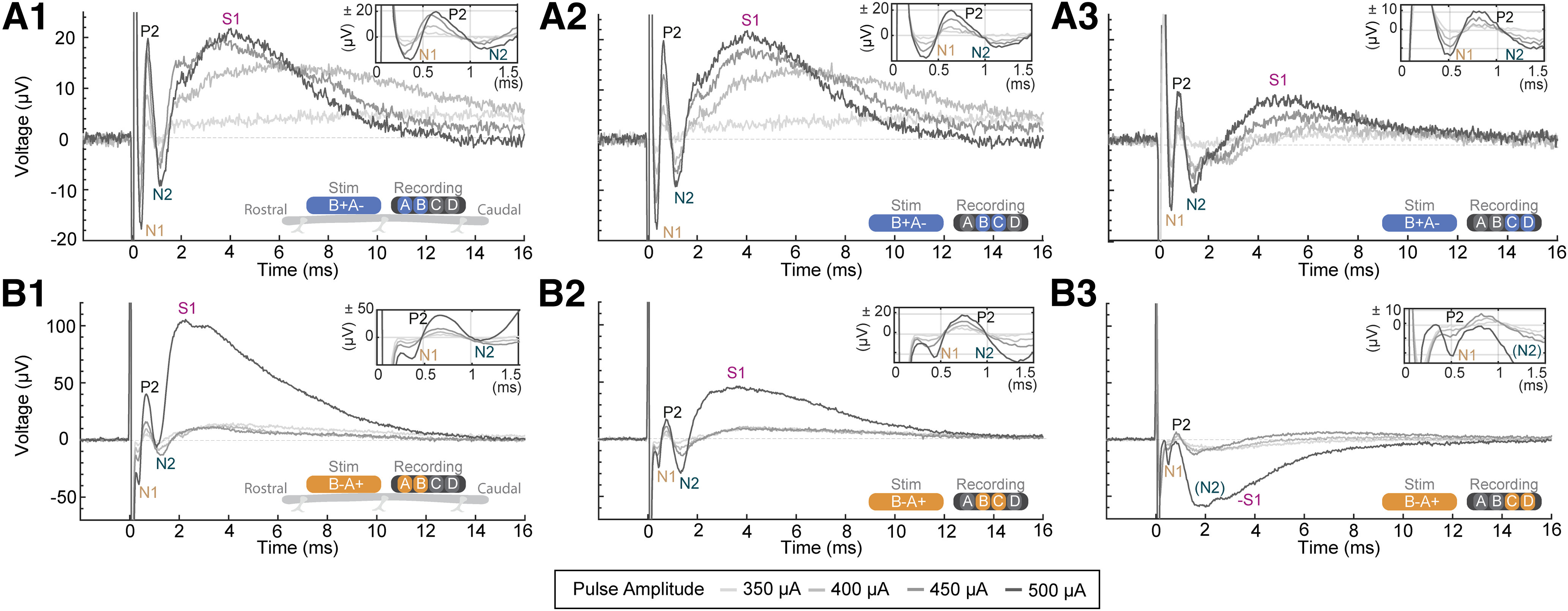
Representative stimulation amplitude versus signal relationships (“dose responses”) of spinal potentials across epidural electrode contracts. The schematic at the lower right side of each panel shows the relative orientation and polarity of the stimulating electrodes (“Stim”), for a proximal cathode (***A***, blue) or proximal anode (***B***, orange) stimulation polarity, as well as the recording electrodes (“Recording”). Each panel shows the average evoked response from 20 low-frequency pulses (40-μs pulse width). The top right inset in each panels magnifies the spinal response from 0 to 1.5 ms. Stimulation intensity is reflecting in gray scale (350 μA, lightest gray; to 500 μA, darkest gray). Identified components of the response are labeled for 500 μA, when observable, as P1, N1, P2, N2, and S1. ***A1***, Dose response for cathode proximal stimulation with A versus B recording. ***A2***, Dose response for cathode proximal stimulation with B versus C recording. ***A3***, Dose response for cathode proximal stimulation with C versus D recording. ***B1***, Dose response for anode proximal stimulation with A versus B recording. ***B2***, Dose response for anode proximal stimulation with B versus C recording. ***B3***, Dose response for anode proximal stimulation with C versus D recording. Please refer to Extended Data Figure 2-1 for the dose response for ECAP and ESAP. ECAP- Evoked Compound Action Potential; ESAP - Evoked Synaptic Activity Potential; P1, N1, P2 - ECAP waveforms; S1- ESAP waveform. The sign ‘-’ indicates the inversion of the waveform (-S1). *Figure Contributions*: Mahima Sharma and Vividha Bhaskar performed the experiments. Mahima Sharma and Vividha Bhaskar analyzed the data. Nigel Gebodh contributed graphics.

**Figure 3.**
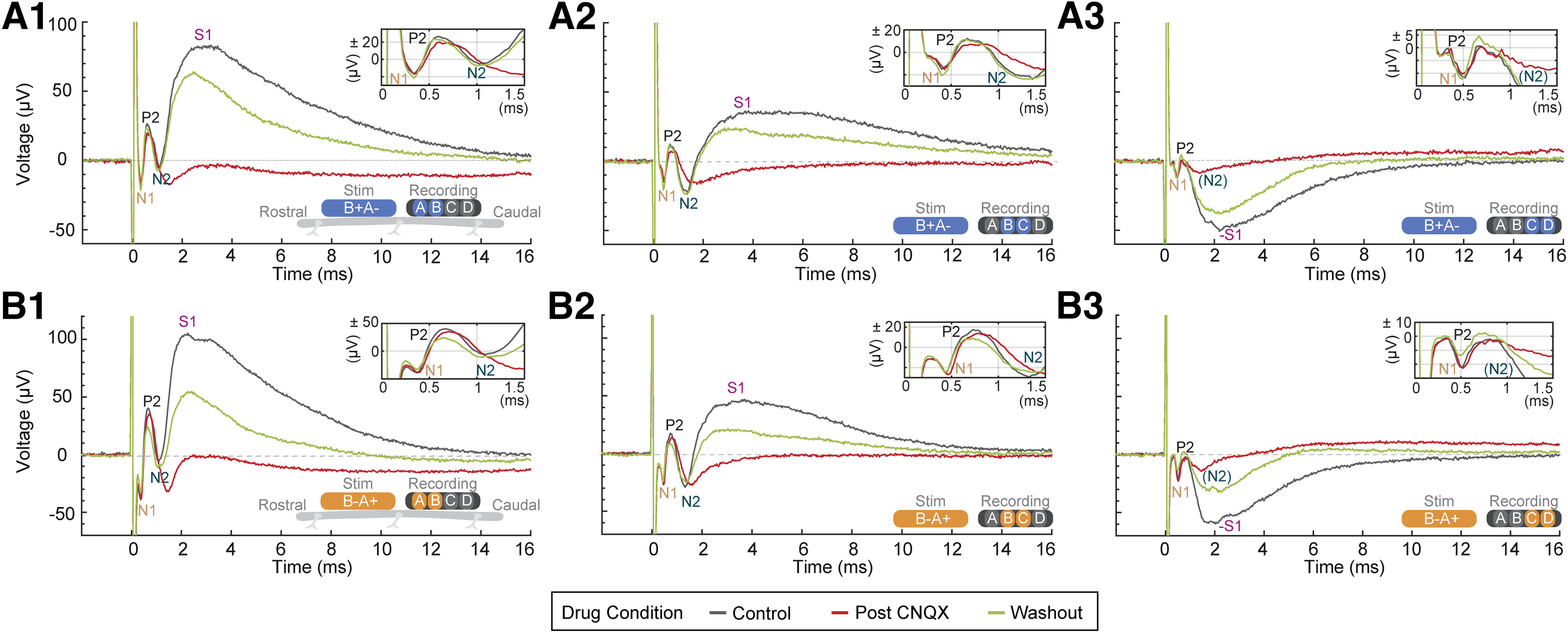
Dependence of evoked epidural spinal waveform on glutamatergic synapses. CNQX, a competitive AMPAR antagonist (1 mm, 100 μl) was applied to the superficial spinal cord surface. Evoked response are recoded before (control, gray), during (after CNQX, red), and after washout (green) of CNQX. The schematic at the lower right side of each panel shows the relative orientation and polarity of the stimulating electrodes (“Stim”), for a proximal cathode (***A***, blue) or proximal anode (***B***, orange) stimulation polarity, as well as the recording electrodes (“Recording”). Each panel shows the average evoked response from 20 pulses (40-μs pulse width). The top right inset in each panels magnifies the spinal response from 0 to 1.5 ms. Stimulation intensity was fixed across all panels. Identified waveform components of the response are labeled for the control condition, when observable, as P1, N1, P2, N2, and S1. ***A1***, CNQX effects for cathode proximal stimulation with A versus B recording. ***A2***, CNQX effects for cathode proximal stimulation with B versus C recording. ***A3***, CNQX effects for cathode proximal stimulation with C versus D recording. ***B1***, CNQX effects for anode proximal stimulation with A versus B recording. ***B2***, CNQX effects for anode proximal stimulation with B versus C recording. ***B3***, CNQX effects for anode proximal stimulation with C versus D recording. *Figure Contributions*: Mahima Sharma and Vividha Bhaskar performed the experiments. Mahima Sharma and Vividha Bhaskar analyzed the data.

The design of the epidural spinal lead used for recording was the same as the epidural lead for stimulation. The epidural recording lead was connected to a differential AC amplifier (A-M Systems; model 1700) through customized connectors that allowed concurrent 100× amplification of three differential recordings (A-B, B-C, C-D; Fig. 2 and Fig. 3) from the four electrodes of the epidural lead. Signals were high pass filtered at 0.1 Hz and low pass filtered at 5 kHz. Signals were digitized (Cambridge Electronic Design 1401; RRID: SCR_017282), and recorded along with stimulation onset triggers (Cambridge Electronic Design Spike2 software; RRID: SCR_000903) at a sampling rate of 33 333 Hz.

10.1523/ENEURO.0429-22.2023.f2-1 Extended Data Figure 2-1 Dose–response curve for ESAP (S1) and ECAP. Monotonic increase in the amplitudes of ECAP (***A***) and ESAP (S1; ***B***) with increasing stimulation intensity across eight animals. ***C***, ***D***, Both ECAP and ESAP (S1), when normalized to the maximum evoked response, exhibit a monotonic progression with increasing stimulation intensity. ***E***, The evoked ESAP (S1), when normalized to corresponding evoked ECAP, show a nonmonotonic progression with increasing current. Data represented as mean of 20 trials from eight animals (color coded). *Figure Contributions:* Mahima Sharma and Vividha Bhaskar performed the experiments. Mahima Sharma analyzed the data. Download Figure 2-1, TIF file.

#### Multichannel intraspinal recording

**Figure 4.**
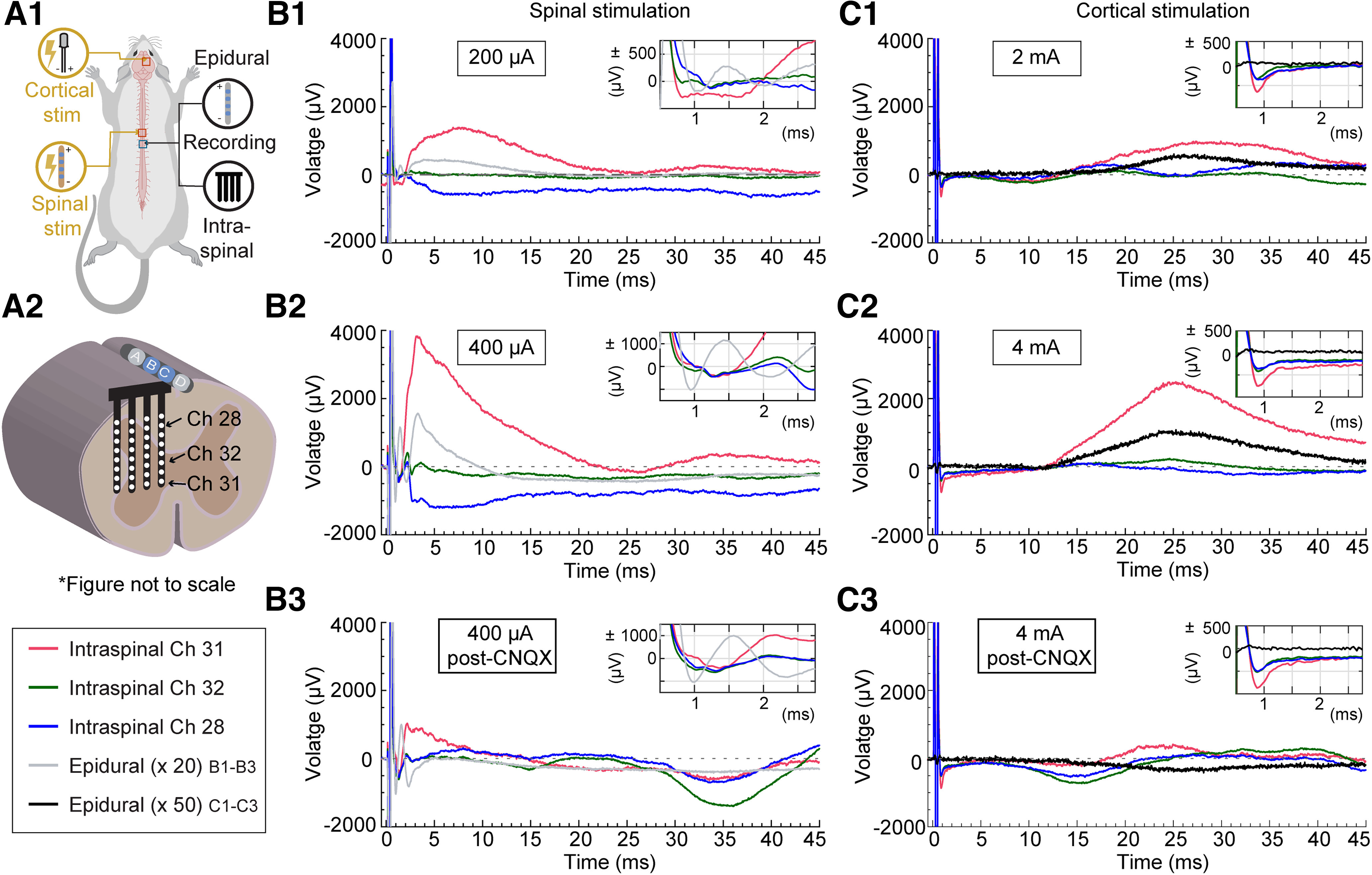
Simultaneous intraspinal and epidural recordings evoked with cortical and epidural spinal stimulation from the same rat. ***A1***, Rat model depicting location of different electrodes (both stimulating and recording, with 4 electrodes in total; Fig. 1*D*). ***A2***, Cross section of spinal cord with intraspinal electrode inserted transversely into the left side at a distance of 350 ± 70 μm from the epidural recording lead placed over the dorsal column with B and C (blue) as active recording electrodes. Intraspinal electrode array allows for recording from different sites: dorsal sector (Channel 28, blue), intermediate sector (Channel 32, black; intermediate sector), and upper ventral sector (Channel 31, red; upper ventral sector). Each panel shows the average evoked response from stimulation with 20 low-frequency pulses (200-μs pulse width) during different current intensities and conditions. ***B***, Epidural spinal stimulation at (***B1***) 200 μA, (***B2***) 400 μA, (***B3***) 400 μA, 15 min after CNQX. Epidural recordings (gray) are magnified 20 times for better comparison. The top right insets in all panels show the spinal response from 0.5 to 2.7 ms after initiation of stimulation artifact. ***C***, Epidural cortical stimulation at (***C1***) 2 mA, (***C2***) 4 mA, (***C3***) 4 mA, 15 min after CNQX. Epidural recordings (black) are magnified 50 times for better comparison. The top right insets in all panels show the spinal response from 0.5 to 2.7 ms after initiation of stimulation artifact. CNQX- a competitive AMPAR antagonist.*Figure Contributions*: Mahima Sharma and Lillian Yang performed the experiments. Mahima Sharma and Nigel Gebodh analyzed the data.

Extracellular, local field potentials (LFPs) were recorded from the L4/L5 spinal segment using a four-shank, 32-channel silicon probe (A4x8-5mm-200–400-177-A32; NeuroNexus; Fig. 1*E* and Fig. 4) optimized for monopolar recording, as previously described (Song and Martin, 2017; Amer et al., 2021). The NeuroNexus probe was inserted transversely into the left side of the spinal cord (contralateral to the cortical stimulation electrode; Fig. 1*A*) to a depth of 1.7 ± 0.2 mm from the pial surface, as measured by the initial pial contact with the electrode array. Electrode depth was controlled using a 100-μm resolution electrode manipulator (David Kopf Instruments). The lateral-right shank of the probe was positioned 350 ± 70 μm from the midline, and the electrode array was lowered, following dura and pia puncture. Each shank was 400 μm apart, with eight recording sites, each with a site area of 177 μm^2^, located 200 μm apart (Fig. 1*E*). Electrode impedance ranged from 500 to 700 kΩ. Electrode positioning was carefully performed in all animals under a dissecting microscope to minimize surface dimpling and electrode position differences among animals.

A unity gain head-stage was connected via flexible cables (A32-OM32 connector; Neuronexus) to the spinal recording array. The broadband signals were amplified (1000×) and low-pass filtered (0.1–300 Hz) for LFP recordings (OmniPlex-D system; Plexon; RRID: SCR_014803). The LFPs were digitized at a sampling frequency of 1 kHz and offline 60-Hz notch-filtered (iirnotch, MATLAB 2021b, MathWorks).

#### EMG recording

EMG was recorded from left tibialis anterior (TA) muscles in the hindlimb or the left *longissimus thoracis* (LT) muscles in the trunk to assay motor evoked potentials (MEPs) and to determine the timing of evoked epidural responses in relation to the MEPs. To record the EMG, percutaneous nickel-chrome wire electrodes were prepared by deinsulating 1 mm of the tip on each wire and folding the wire back on itself from the exposed tip (“hook electrode”). The wires were inserted into the belly of tibialis anterior or longissimus thoracis muscles using a 30-gauge hypodermic needle and were positioned ∼2 mm apart from each other. Electrode location was verified by delivering a series of biphasic pulses to the dorsal epidural SCS electrode observing an EMG response through the inserted EMG electrodes.

**Figure 5.**
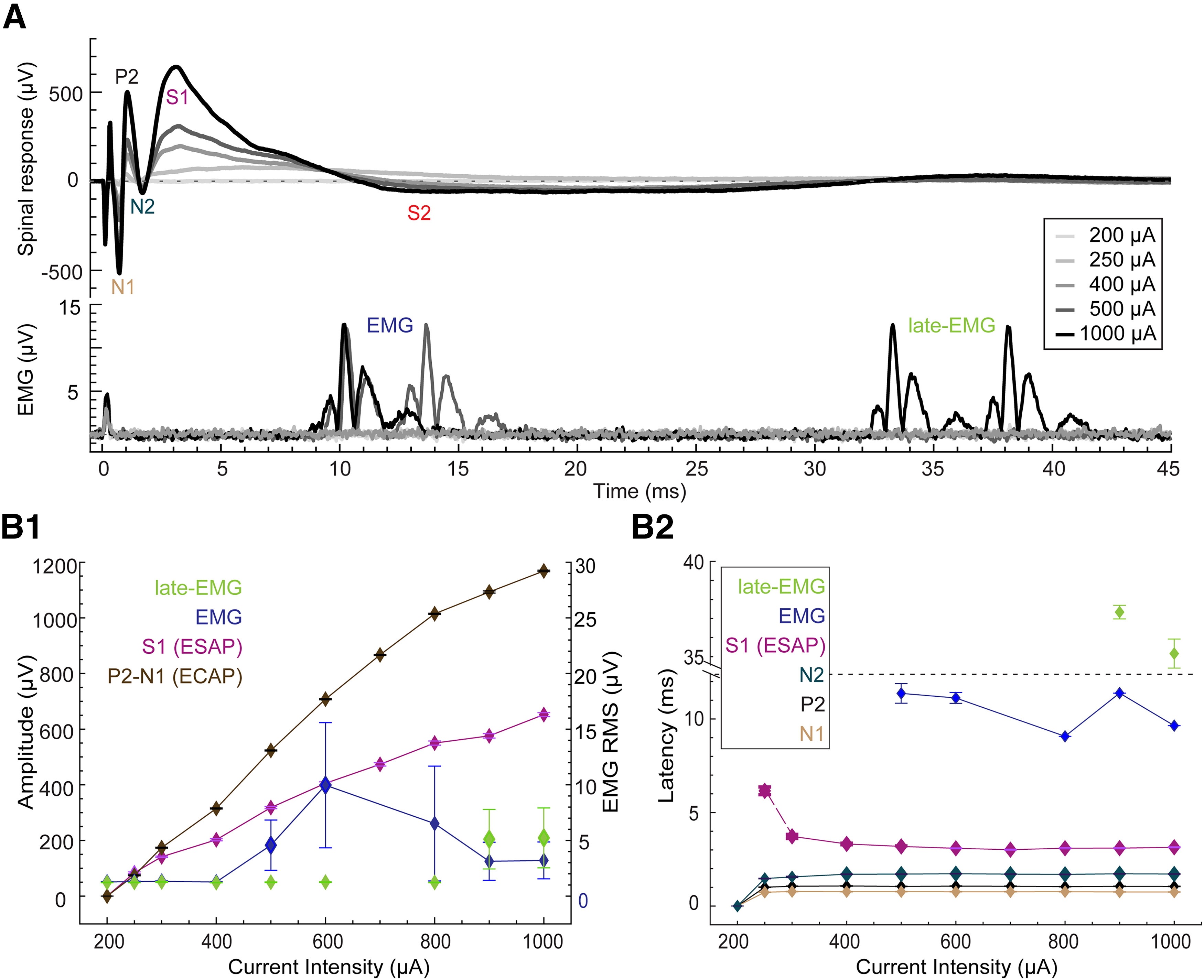
Dose response for the spinal and the hindlimb EMG recordings. ***A***, Average evoked spinal response [ECAP, evoked synaptic activity potential ESAP (S1, S2); upper panel] and average evoked EMG (tibialis anterior hindlimb muscle; lower panel) response from 20 low-frequency pulses of epidural stimulation (Fig. 1*A*) of spinal cord at varying current intensities, ranging from 200 to 1000 μA, in a representative single rat. Identified waveform components of the spinal response are labeled for 1000 μA, when observable, as P1, N1, P2, N2, S1, and S2 (ESAP). ESAP (S1) becomes more distinct with increasing current intensity. Two types of EMG responses were observed: those occurring before 20 ms, referred to as EMG (blue), and those occurring after 30 ms, referred to as late-EMG (green). ***B1***, Dose–response curve for the ECAP (brown), ESAP (S1; pink), EMG (blue) and late-EMG (green) responses. The left axis represents the amplitude for ECAP and ESAP (S1); the right axis represents the RMS for EMG responses. Each data point represents mean ± SEM (20 trials). ***B2***, Latencies plotted as a function of current intensities. N1 (brown), P2 (black), N2 (dark green), and S1 (ESAP, pink). Latency for the EMG and late-EMG is defined as the latency of the first peak in the EMG/late-EMG response. Data represented as mean ± SEM. ECAP- Evoked Compound Action Potential; ESAP - Evoked Synaptic Activity Potential; P1, N1, P2 - ECAP waveforms; S1 & S2 - ESAP waveforms. *Figure Contributions*: Mahima Sharma and Vividha Bhaskar performed the experiments. Mahima Sharma analyzed the data.

For hindlimb EMG experiments, the presence of MEPs was monitored in response to the train of pulses to the contralateral M1 HLA or dorsal spinal cord. For trunk EMG experiments, the presence of MEPs was monitored in response to the train of pulses through the epidural electrodes. EMG data were continuously acquired with a differential AC amplifier system (A‐M Systems, Model 1700), amplified at a gain of 1000, high pass filtered at 300 Hz and low pass filtered using second order Butterworth filter at 5 kHz (hindlimb EMG from TA) or 20 kHz (trunk EMG from LT). Signals were digitized using a CED 1401 data acquisition system (Cambridge Electronic Design), and recorded using Spike2 software (Cambridge Electronic Design) at a sampling rate of 33,333 Hz, and rectified. EMG threshold is defined as the current amplitude that evoked responses with an amplitude greater than three times the SD of the background signal level in 20% of the trials. EMG amplitudes are reported as root mean square (RMS) over 1–20 ms for initial hindlimb EMG, 25–45 ms for late hindlimb EMG and 1–9.9 ms for trunk EMG (Fig. 5).

### Drug administration

To confirm the synaptic nature of the delayed wave component of the spinal potential, a competitive AMPA receptor (AMPAR) antagonist, 6-cyano-7-nitroquinoxaline-2,3-dione (CNQX; 100 μl, 100 μm to 1 mm) was applied to the exposed spinal cord where the dura and pia were punctured at the L4/L5 spinal segment. 10 mm stock solutions of CNQX disodium salt hydrate (Sigma-Aldrich, catalog #C239) were prepared by dissolving in 30% DMSO solution. Stocks were then diluted in warm saline (37°C) to get the desired concentration of CNQX. The final concentration of DMSO in the solution never exceeded 0.1%. To control for the vehicle used in the experiments, warm saline solution (100 μl) was applied to the same site 20 min before the CNQX application. The vehicle-only experiment was done once or twice before the CNQX application, and sequential administration of CNQX enabled animals to be used as their own controls. The experiments with consistent evoked responses between the two saline conditions or the ones with no more than 40% change in the evoked response with saline application, were included for further analyses. After ∼20 min of drug application, warm saline (200 μl) was applied to wash out the CNQX.

**Figure 6.**
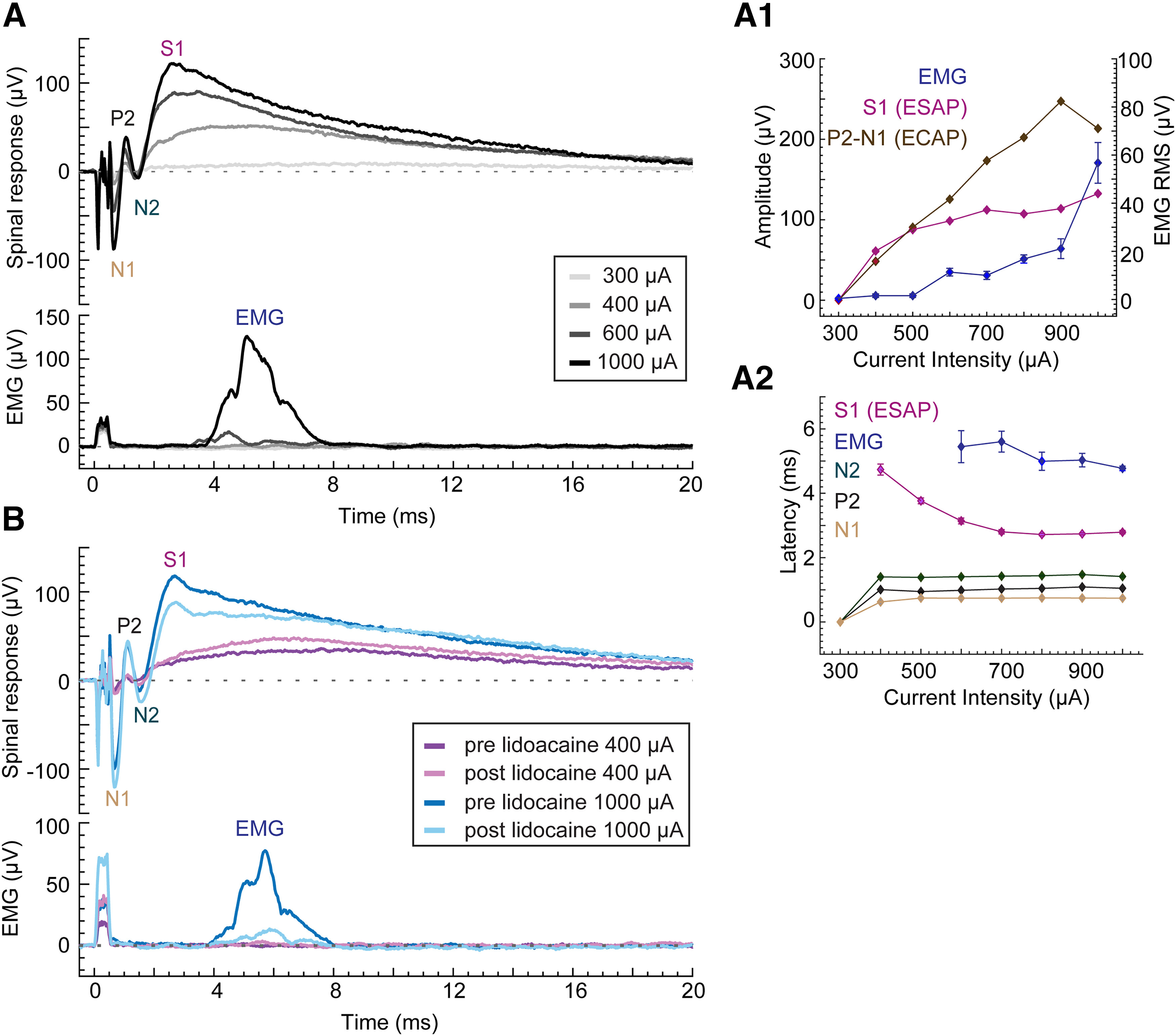
Dose response for the spinal and the trunk EMG recordings. ***A***, Average evoked spinal response [ECAP, evoked synaptic activity potential ESAP (S1); upper panel] and average evoked trunk EMG (LT muscle; lower panel) response from epidural stimulation of spinal cord at varying current intensities, ranging from 200 to 1000 μA, with a train of 40 pulses (40-μs pulse width, 1 Hz) in a representative single rat. Identified waveform components of the spinal response are labeled for 1000 μA, when observable, as P1, N1, P2, N2, S1, and S2 (ESAP). ESAP (S1) becomes more distinct with increasing current intensity. ***A1***, Dose–response curve for the ECAP (brown), ESAP (S1; pink), EMG (blue), and late-EMG (green) responses. The left axis represents the amplitude for ECAP and ESAP (S1); the right axis represents the RMS for EMG responses. Each data point represents mean ± SEM (40 trials). ***A2***, Latencies plotted as a function of current intensities. N1 (brown), P2 (black), N2 (dark green), and S1 (ESAP, pink). Latency for the trunk EMG is defined as the latency of the first peak in the EMG response. Data represented as mean ± SEM (40 trials). ***B***, Same as ***A***, but only at two SCS current intensities: 400 and 1000 μA, prelidocaine and 20 min after lidocaine in the same rat. ECAP- Evoked Compound Action Potential; ESAP - Evoked Synaptic Activity Potential; P1, N1, P2 - ECAP waveforms; S1- ESAP waveform.*Figure Contributions*: Mahima Sharma performed the experiments and analyzed the data.

To assess the relationship between EMG and ESAP response further, the local anesthetic lidocaine (Henry Schein Medical, 0.5%, 0.1 ml) was injected into the right and left LT muscles. In lidocaine experiments, the trunk EMG assay, as previously described, was repeated using epidural stimulation pulses with intensities putatively below and above EMG threshold immediately before and 20 min after lidocaine application (Fig. 6).

### Data analysis

All signal processing in this study was performed with MATLAB (2021b, MathWorks; RRID: SCR_001622). Raw data were exported from Spike2 to MATLAB for analysis and following the offset correction, the average responses from the 20 or 40 trials (subset of animals where trunk EMG was recorded) were computed for each experiment. To characterize and compute the ECAP amplitude and the delayed wave, the positive peaks (P2, S1; Figs. 2, 3, 5, 6) and the negative troughs (N1, N2) were located, and their amplitude and latencies were computed. The ECAP amplitude was always calculated as the peak-to-peak amplitude (voltage) between the first negative phase (N1) and the second positive peak (P2) of the ECAP. Latency was measured as the time from the onset of stimulus artifact to the onset of the first deflection of the potential.

EMG responses were rectified and root mean square (RMS) and latency calculated. Data for different parameters of ECAP components, delayed wave and EMG are reported as mean ± SEM. Threshold for ECAP is defined as the minimum current amplitude that evoked responses in 95% of the trials that exceeded 10 μV. Threshold for the delayed wave component (S1) is defined as the minimum current amplitude that evoked responses in 95% of the trials that exceeded 25 μV.

### Statistical analysis

Data were tested for normality using one sample Kolmogorov–Smirnov test (kstest, MATLAB) or Shapiro–Wilk test (swtest, MATLAB). Statistical comparisons included parametric tests (*t* test, MATLAB) or nonparametric tests (Wilcoxon signed-rank test, signrank, MATLAB) for datasets with normal or non-normal distribution, respectively. One-way ANOVA (anova1, MATLAB) was used to compare the percent change in amplitudes and latencies of ECAP and S1 across low-frequency or tonic (50 Hz) SCS. A Bonferroni’s *post hoc* correction was used for multiple comparisons. Error bars indicate mean ± SEM, *p* values < 0.05 were considered significant. Further details on the comparisons made and statistical tests used are presented in Table 1. Custom MATLAB code that was used for analyses available on request.

**Table 1:** Summary of statistical analysis used to characterize the Evoked Synaptic Activity Potential (ESAP)

Figure	Measurement	Comparison	*N*	Statistical test	Results
	S1 (ESAP) amplitude	Control vs CNQX	14	Wilcoxon signed-rank test	*p* = 0.0001
	ECAP amplitude	Control vs CNQX	14	Wilcoxon signed-rank test	*p* = 0.0562
	Spinal stimulation evoked S1 (ESAP) latency	Intraspinal vs epidural response	10	Paired sample *t* test	*t*_(9)_ = 1.35, *p* = 0.38
	Motor cortical stimulation evoked S1 (ESAP) latency	Intraspinal vs epidural response	10	Paired sample *t* test	*t*_(9)_ = 1.73, *p* = 0.15
	Current thresholds	S1 vs EMG	9	Paired sample *t* test	*t*_(8)_ = 6.04, *p* < 0.0001
	Latencies	S1 vs EMG	9	Paired sample *t* test	*t*_(8)_ = 15.7, *p* < 0.0001
7*A*	S1 (ESAP) amplitude during 50-Hz SCS	Increasing stimulation pulse count (1–20)	5	One-way ANOVA	*F*_(19,80)_ = 6.5162, *p* = 8.4244e-10
7*A*	S1 (ESAP) amplitude during 1-Hz SCS	Increasing stimulation pulse count (1–20)	5	One-way ANOVA	*F*_(19,80)_ = 1.3284, *p* = 0.1898
7*B*	ECAP amplitude during 50-Hz SCS	Increasing stimulation pulse count (1–20)	5	One-way ANOVA	*F*_(19,80)_ = 1.2822, *p* = 0.2188
7*B*	ECAP amplitude during 1-Hz SCS	Increasing stimulation pulse count (1–20)	5	One-way ANOVA	*F*_(19,80)_ = 0.3226, *p* = 0.9964
7*D*	S1 (ESAP) latency during 50-Hz SCS	Increasing stimulation pulse count (1–20)	5	One-way ANOVA	*F*_(19,80)_ = 0.75, *p* = 0.7579
7*C*	S1 (ESAP) latency during 1-Hz SCS	Increasing stimulation pulse count (1–20)	5	One-way ANOVA	*F*_(19,80)_ = 0.23, *p* = 0.9996
7*D*	ECAP (N1) latency during 50-Hz SCS	Increasing stimulation pulse count (1–20)	5	One-way ANOVA	*F*_(19,80)_ = 0.02, *p* = 1
7*C*	ECAP (N1) latency during 1-Hz SCS	Increasing stimulation pulse count (1–20)	5	One-way ANOVA	*F*_(19,80)_ = 1.73, *p* = 0.049
7*D*	ECAP (P2) latency during 50-Hz SCS	Increasing stimulation pulse count (1–20)	5	One-way ANOVA	*F*_(19,80)_ = 0.06, *p* = 1
7*C*	ECAP (P2) latency during 1-Hz SCS	Increasing stimulation pulse count (1–20)	5	One-way ANOVA	*F*_(19,80)_ = 0.95, *p* = 0.5225

## Results

### Characterization of ECAP and S1 SCS evoked responses

We systematically explored the waveform of potentials evoked by a rostrally-positioned spinal epidural lead and recorded by a caudally-positioned spinal epidural lead (*n* = 39). Stimulation was delivered in trains of single biphasic pulses (Fig. 1*A*) through varied stimulation or recording electrodes, and stimulation polarities and intensities (Fig. 1). Representative responses from one animal are shown (Fig. 2). Stimulation could evoke a characteristic multiphasic early response, reported in human and other rodent studies, including a P1, N1, P2, and N2, which together form the ECAP, at a mean threshold of 237 ± 15 μA (*n* = 39). The earliest component of the ECAP, the positive P1, was in some cases obscured by the stimulus artifact and was easiest to resolve with increasing interelectrode distance (e.g., recording from electrode position C vs D) and with proximal anode stimulation polarity (Fig. 2*B3*). The average P1 latency was 0.32 ± 0.02 ms (*n* = 39). The next component was the negative N1 (0.56 ± 0.02 ms, *n* = 39), followed by the positive P2 (0.87 ± 0.03 ms, *n* = 39), and negative N2 (1.35 ± 0.04 ms, *n* = 39). The relative latency of each of these components increased with distance from stimulation to recording electrode (Fig. 2, left to right columns) and when the distance from the proximal cathode was changed by reversing stimulation polarity, consistent with axonal propagation (Fig. 2*A* vs *B* row). However, the polarity of N1, P2, and N2 did not invert with the stimulation artifact and was consistent across recording electrode pairs. In a minority of experiments, N2 was composed of dual peaks (data not shown). Taken together, these recordings indicate that epidural stimulation was functional and could evoke ECAP responses indicative of dorsal column activity consistent with prior observations (Cedeño et al., 2022; Dietz et al., 2022).

We further identified a “S1” wave with a latency longer than N2 and a mean threshold of 307 ± 16 μA (*n* = 39). Both ECAP and ESAP amplitude increased monotonically with increasing current intensities, but the ratio of ESAP to ECAP neither increased monotonically nor stayed at the same value across all stimulation amplitudes (Extended Data Fig. 2-1*E*), supporting a distinct origin for the ESAP. Electrode position and stimulation dose (intensity, polarity) response testing also identified distinct features of the S1-wave versus the other waveform components. The S1-wave appeared after N2 (2.99 ± 0.15 ms, *n* = 39), although the initiation of the S1-wave appeared to overlap with the N2. In addition to starting later than the ECAP components, the S1-wave has a longer duration (6.34 ± 0.04 ms) as compared with ECAP (1.01 ± 0.02 ms, signed-rank *p* < 0.0001, *n* = 39), with a distinctive peak at higher current intensities (example in Fig. 6). Furthermore, unlike the ECAP, which retained a relatively consistent amplitude and morphology across differential pairs, the relative amplitude of S1-wave and ECAP changed with stimulation to recording electrode distance (Figs. 2*A1* vs *A2* vs *A3*, *B1* vs *B2* columns) and could invert at caudal electrode sites (Fig. 2*B3*). In such inversions, the polarity of the S1-wave was the same as N2. Inverting stimulation polarity altered S1 morphology on a given recording pair but did not necessarily invert the S1-wave, suggesting spatial dependence of S1 on the location of the stimulating cathode but ruling against S1 being a stimulation artifact (Fig. 2*A* vs *B*). Taken together, these results demonstrate a unique stimulation-response relationship and spatiotemporal profile for S1-waves compared with ECAPs, consistent with a distinct physiological source.

### Synaptic origin of S1-waves (ESAPs)

The dependence of spinal evoked potentials on glutamatergic synaptic transmission was assessed using CNQX (Fig. 3), a competitive AMPAR antagonist (1 mm, 100 μl). Subdural superfusion of CNQX resulted in a significant attenuation of the S1-wave from 52.2 ± 19.1 μV (control, vehicle) to 22.8 ± 10.1 μV (15 min after CNQX, *n* = 14) but did not affect the mean ECAPs (P2-N1: 57.5 ± 15.5 μV, control to 54.8 ± 15.7 μV, 15 min after CNQX). The mean S1-wave amplitude significantly decreased following CNQX (mean decrease of 62.2 ± 6.7%; signed-rank *p* = 0.0001, *n* = 14), whereas the ECAP did not significantly decrease (10.5 ± 5.6%; signed-rank *p* = 0.0562, *n* = 14). Washout with saline (vehicle) resulted in the recovery of the S1-wave amplitude after 45 min (Fig. 3), consistent with the reversible nature of the selective AMPA receptor blockade by CNQX. Given the distinct waveform following CNQX but *not* vehicle alone and strong selectivity for AMPA receptors by CNQX, we conclude that the S1 wave is a type of evoked spinal synaptic response, or “ESAP.” The origin and physiology of ESAPs are analyzed further in the next sections.

### Intraspinal analog of ESAPS

Simultaneous recording of epidural and intraspinal potentials during epidural spinal stimulation (Fig. 4, column B) showed an intraspinal analog of the S1-wave (ESAP) as well as the ECAP [called afferent terminal potentials (ATPS); Contreras-Hernández et al., 2022]. The mean latencies of the S1 peak and its intraspinal analog were 3.11 ± 0.16 ms and 2.78 ± 0.23 ms, respectively (*t*_(9)_ = 1.35, *p* = 0.38, *n* = 10). The intraspinal response was spatially specific and could reverse in polarity- consistent with intraspinal sinks/source. The intraspinal analog of the S1-wave was also suppressed by CNQX (Fig. 4*B3*), further supporting the intraspinal synaptic origin of ESAPs. A later slow wave potential was also observed in both epidural (10–30 ms) and intraspinal recordings (15–30 ms). Taken together, these intraspinal recordings suggest local sites of sinks/sources underlying the S1 wave, consistent with spinal synaptic currents.

We further considered the nature of the S1-waves and their intraspinal analog by evoking responses by epidural motor cortex stimulation. The spinal response to motor cortex stimulation reflects intraspinal synaptic excitatory potentials (Amer et al., 2021; Zinger et al., 2013). Motor cortex stimulation produced a response recorded using epidural electrodes that was delayed and prolonged compared with spinal-stimulation evoked S1-waves (Fig. 4, column C), with a mean latency of 23.33 ± 0.90 ms. Intraspinal recordings reflect a similar waveform and latency of 22.53 ± 0.85 ms (*t*_(9)_ = 1.73, *p* = 0.15, *n* = 10), with spatial sensitivity (consistent with local intraspinal sinks/sources). Cortical evoked responses were abolished by superfusion with CNQX (Fig. 4*C3*). These results using cortical stimulation confirm intraspinal (excitatory) signals can be detected with bipolar epidural cylindrical electrodes. The general correspondence between spinal responses to cortical versus spinal stimulation should not imply the same synapses are active (only that they may overlap in location and type).

### Ruling out EMG as an explanation for ESAP (S1)

The SCS current threshold, latencies, and behavior of the S1 ESAPs were compared with hindlimb/trunk EMG responses to assess whether or not S1 was directly related to EMG. In the subset of 9 animals from which both epidural and hindlimb EMG responses were recorded, mean S1 thresholds were significantly lower than hindlimb EMG thresholds (S1: 328 ± 28 μA vs EMG: 694 ± 72 μA, ratio: 0.49 ± 0.04; *t*_(8)_ = 6.04, *p* < 0.0001, *n* = 9). The current threshold for eliciting a visually-observed motor response was approximately double the hindlimb EMG threshold. As noted above, both ECAP and ESAP amplitude increased monotonically with stimulation current intensity (Fig. 5*B1*), but not ECAP to ESAP ratio (Extended Data Fig. 2-1*C*).

With respect to latency, two distinct hindlimb EMG responses were evoked: a first EMG (∼9 ms) and a “late-EMG” (∼35 ms). Hindlimb EMG responses were stimulation-amplitude dependent. Across animals, ESAPs emerged gradually with increasing SCS amplitude (Extended Data Fig. 2-1*B*) and significantly earlier than first EMG (ESAP = 3.06 ± 0.12 ms; EMG = 9.37 ± 0.43 ms, *t*_(8)_ = 15.7, *p* < 0.0001, *n* = 9) and did not overlap in time with either the first or late EMG. The mean difference between the latencies for ESAP, measured at the S1 peak, and first hindlimb EMG, measured at the first rising edge of the signal, was 6.31 ± 0.4 ms (*n* = 9).

In a separate experiment series, both epidural responses and trunk EMG (LT muscles) were recorded from a subset of six animals (Fig. 6). No EMG was observed in one animal at up to the highest current amplitude tested (2000 μA). The mean S1 thresholds were significantly lower than trunk EMG thresholds (S1: 360 ± 40 μA vs EMG: 1320 ± 239 μA, ratio: 0.32 ± 0.09 *t*_(4)_ = 4.19, *p* = 0.01, *n* = 5). The current threshold for eliciting a visually-observed motor response was a further ∼1.7× the trunk EMG threshold. The mean difference between the latencies for ESAP, measured at the S1 peak (2.37 ± 0.16 ms), and the initiation of the (5.42 ± 0.8 ms), was 3.05 ± 0.8 ms (*t*_(2)_ = 3.7, *p* = 0.06, *n* = 3; Fig. 6*A2*). After injecting the local anesthetic lidocaine into the left and right LT muscle, EMG responses decreased 80 ± 1% from 18.5 ± 6 μV RMS (prelidocaine) to 3.6 ± 1 μV RMS (20 min after lidocaine; Fig. 6*B*). There was no manifest change in the S1 peak amplitude (*n* = 3): 269 ± 97 μV (prelidocaine), 204 ± 84 μV (20 min after lidocaine). In summary, the higher thresholds and longer latencies of EMG responses compared with ESAPs, as well as distinct waveforms and response to local anesthetic injection, further corroborate that ESAPs are not measuring muscle responses (i.e., ESAPS are not myogenic).

### Effects of SCS frequency on ECAPs versus ESAPs (S1)

**Figure 7.**
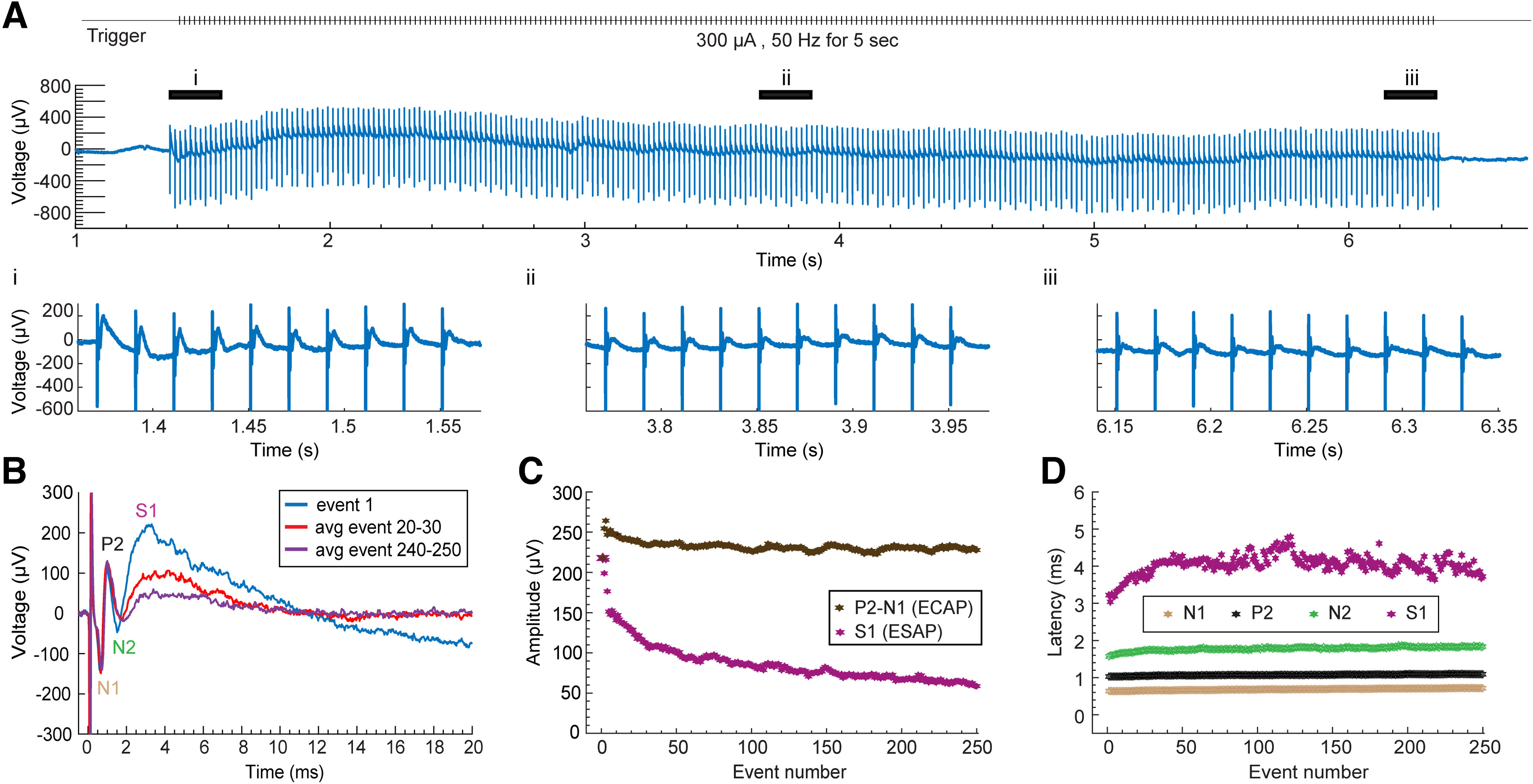
Tonic stimulation (50 Hz) evoked ECAP and ESAP (S1). ***A***, Evoked responses from the tonic stimulation of epidural spinal cord at 300 μA (40-μs pulse width, 250 stimulation events) Trigger is shown at the top. The black lines at top correspond to early (i), middle (ii), and late (iii) events shown at the bottom. The ESAP (S1) is distinctly recognizable in (i), attenuates in (ii) and (iii). ***B***, Representative traces of the evoked response comprising of ECAP and ESAP (S1) from event 1 (blue), averaged responses from event 20 to event 30 (red), and those from event 240–250 (purple) show a gradual decrease in the ESAP (S1) amplitude with increasing events. ***C***, Comparison of the ESAP (S1) amplitude (pink) between events. Moving average of four events is computed to smoothen the data for the amplitude of ESAP (S1) from event 7 and the amplitude of ECAP (P2-N1, brown) from event 4 onwards. Rest of the data are represented in absolute values. The ESAP (S1) amplitude drops while the ECAP amplitude stays consistent except for the first three events. ***D***, Latencies for N1, P2, N2, and S1, plotted as a function of event. Moving average of four events is computed to smoothen the data for the latencies of ESAP (S1, pink) and ECAP (N1, brown; P2, black, N2, green) from event 4 onwards. ECAP- Evoked Compound Action Potential; ESAP - Evoked Synaptic Activity Potential; P1, N1, P2 - ECAP waveforms; S1- ESAP waveform.*Figure Contributions*: Mahima Sharma performed the experiments and analyzed the data.

**Figure 8.**
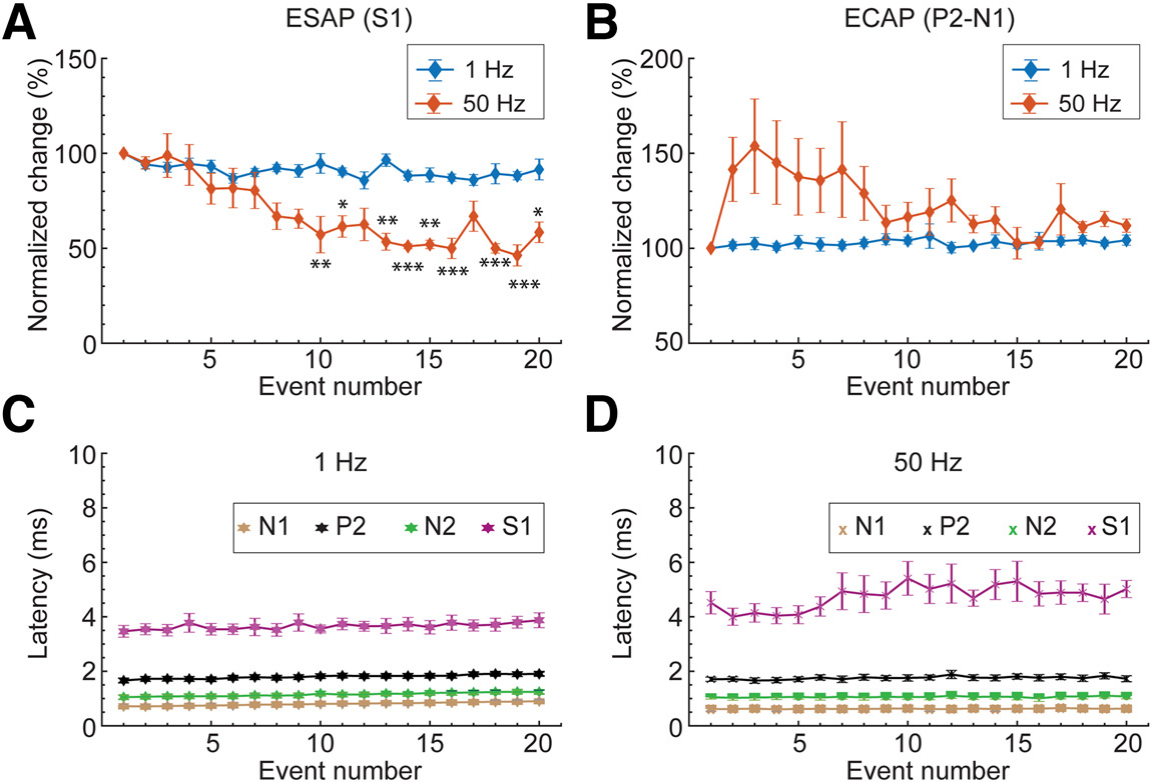
Sensitivity of ESAP (S1) and ECAP to 1 Hz versus Tonic (at 50 Hz) SCS. ***A***, Comparison of the evoked ESAP (S1) responses from 1-Hz stimulation events (blue, Fig. 1*A*) versus 50-Hz SCS events (orange). Evoked ESAP responses at different stimulation events (1–20) are reported as the percentage of the ESAP value at the first event. ***B***, Comparison of the evoked ECAP responses from 1-Hz stimulation (blue) versus 50-Hz stimulation (orange). Evoked ECAP values at different stimulation events (1–20) are reported as the percentage of the ECAP value at the first event. ***C***, Latencies for evoked ECAP and ESAP: N1 (brown), P2 (black), N2 (green), and S1 (pink) for 1-Hz stimulation (*n* = 5). ***D***, Latencies for evoked ECAP and ESAP: N1 (brown), P2 (black), N2 (green), and S1 (pink) for 50-Hz stimulation. Data represented as mean ± SEM (*n* = 5). Statistical power using Bonferroni *post hoc* test comparing percent change in amplitudes at event 1 with those at rest of the events assumed at *p < 0.05, **p < 0.01, ***p < 0.001. ECAP- Evoked Compound Action Potential; ESAP - Evoked Synaptic Activity Potential; P1, N1, P2 - ECAP waveforms; S1- ESAP waveform.*Figure Contributions*: Mahima Sharma performed the experiments and analyzed the data.

We evaluated the dependence of ECAPs and S1 ESAPs on the frequency of spinal cord stimulation (SCS) by evoking responses at 50-Hz SCS (Fig. 7). Responses are shown as a function of stimulation pulse index (event) to allow for comparison with evoked responses at low-frequency (1 Hz) SCS. One-way ANOVA revealed a significant decrease in the S1 amplitude (Fig. 7*B*,*C*) with increasing stimulation pulse count during 50-Hz SCS (*F*_(19,80)_ = 6.5162, *p* < 0.001), unlike during 1-Hz SCS (*F*_(19,80)_ = 1.3284, *p* = 0.1898; Fig. 8*A*). *Post hoc* Bonferroni’s multiple comparison test demonstrated a significant change in S1 amplitude during 50-Hz SCS starting from event 10 (*p* = 0.0082), with an average 51% decrease by event 14 (*p* = 0.0007, *n* = 5). In contrast, despite a nonsignificant trend toward facilitation in the first 9 events during 50-Hz SCS, there was no consistent significant change in mean ECAP amplitudes across stimuli events (50 Hz: *F*_(19,80)_ = 1.2822, *p* = 0.2188; 1 Hz: *F*_(19,80)_ = 0.3226, *p* = 0.9964; *n* = 5; Fig. 8*B*). Furthermore, there was no significant change in the latencies of ECAP and S1 across stimulus events for either 1- or 50-Hz stimulation (*F*_(19,80)_ < 1, *p* > 0.05, *n* = 5; Fig. 8*C*,*D*). Therefore, S1 ESAPs amplitude are distinctly sensitive to SCS frequencies up to 50 Hz in contrast to ECAPs, suggesting a slower adapting response underlying S1 ESAPs. No visual motor responses were observed at the thresholds analyzed.

## Discussion

### Features and detection of S1 ESAPs

We show that SCS can evoke an epidural electrophysiological response termed “ESAPs” reflecting spinal synaptic currents, that are distinct (in dose response, and etiology) to well-characterized ECAPs from dorsal column axons. The observations of the S1 wave supporting this are:

- Delayed onset and time course compared to ECAPs and N2 (Figs. 2, 5);
- Segmental sensitivity and specificity (Fig. 2);
- Amplitude changes and possible inversion in signal polarity across segments, in contrast to relatively fixed waveform ECAPs (Fig. 2);
- Monotonic sensitivity to stimulation intensity, but with distinct dose response from ECAPs (Fig. 2; Extended Data Fig. 2-1);
- Sensitivity to stimuli polarity (Fig. 2), but no direct correlation in terms of response polarity reversal, ruling against a stimulation artifact;
- Threshold below EMG threshold (Figs. 5*B1*, 6*A1*), peaking before EMG initiation (Figs. 5*B2*, 6*A2*) and occurring independently of EMG (e.g., absence during “late-EMG”; Fig. 5*A1*), which along with ESAP spatiotemporal features rules out a myogenic origin;
- Suppression by glutamatergic-synaptic antagonist (Figs. 3, 4), without concurrent block of ECAPs;
- Diminishment (but still detectable) with frequency increases from 1 to 50 Hz, in contrast to relative stability of ECAPs (Fig. 8);
- Intraspinal correlate consistent with grey-matter origin, that is also suppressed to by a glutamatergic-synaptic antagonist (Fig. 4*B*);
- Epidural electrodes measure responses to cortical stimulation, confirming detectability of spinal synaptic activity (Fig. 4*C*).

The ESAPs spatial distribution remains to be fully characterized as this is key to understanding the etiology of S-waves and their relevance to pain and sensory processing. Since the detection of the S1-wave is spatially constrained and dependent on the stimulation site, we suspect ESAPs reflect local connectivity (circuits), in contrast to the broadly detectable, propagating ECAPs that represent activity along dorsal column axons of passage. Thus, the absence of observable ESAPs in prior studies (Parker et al., 2013) despite visible ECAPs can be explained by variation in electrode placement. In addition, the S1-wave latency and duration approximates or exceeds the conventional SCS rates (interpulse intervals corresponding to 50- to 100-Hz frequency), that may explain why ESAPs were previously missed; along with the routine explanation that reported ECAP responses are analyzed in a limited (∼3 ms) poststimulation pulse time window (Parker et al., 2012; e.g., Figs. 2, 3, insets). We further distinguish between ESAPs and multiphasic ECAPs, as reported previously (Chakravarthy et al., 2020) and observed in our series (data not shown), which reflect multiple dorsal column axon volleys, and so are distinct from S1-waves.

In contemporary SCS, limited reports of “late response” spinal potentials suggested their unreliability and/or association with undesired side-effects (Parker et al., 2012; Falowski et al., 2022). We show SCS ESAPs are reliably evoked provided electrodes are positioned at specific sites, and are distinct from EMG. We also distinguish two types of SCS evoked spinal potentials labeled “late response” initiating at either ∼10 ms (Parker et al., 2012) or ∼4 ms (Falowski et al., 2022). In the latter case, it was shown EMGs initiate after the “late response,” ruling out a myogenic origin. Muscle responses from SCS are thought to reflect activation of smaller diameter dorsal root fibers that then produce muscular activity through activation of motor neurons via reflex pathways in the ventral horn (Shils and Arle, 2012). The observation that some SCS conditions (e.g., lateral electrode placement) produce both slow spinal potentials and undesired side effects (EMG, discomfort) is therefore not surprising, and should not be confused with SCS conditions optimized for ESAPs (e.g., midline stimulation electrode at specific distance from recording electrode).

Background supporting the hypothesis that ESAPs reflect local circuit processing are wide-ranging experiments on spinal evoked (cord dorsum) potentials (Maruyama et al., 1982; Shimoji et al., 1982; Yates et al., 1982; Tomita et al., 1996; Ondrejcák et al., 2005); a ∼2-ms “triphasic spike” (attributed to axonal conduction, e.g., ECAPs), ∼5-ms “negative wave” (attributed to synaptic afferents/interneuron activity), and then a tens of millisecond “positive wave” [attributed to synaptic “primary afferent depolarization (PAD)” or presynaptic inhibition] have been reported. That the properties of the ESAP are sensitive to the frequency of applied SCS, with higher frequencies (i.e., 50 Hz) producing adaptation of ESAP but not ECAP, corroborates either an adapting excitatory synaptic mechanism or a result of PAD, i.e., presynaptic inhibition of excitatory afferents, as the underlying mechanism of generation. Indeed, the discovery of SCS was motivated by slow spinal responses (“after-discharges”) in the manifestation and control of pain (Shealy et al., 1970), typically recorded in the form of diffuse field potentials (Shealy, 1966). We caution against inconsistent labeling of evoked spinal responses (e.g., indexing positive and negative polarity waves) and hence adopted “S-wave” here, reflecting synaptic origin (and indeed reversible polarity).

### Origin and significance of S1 ESAPs

The occurrence of ESAPs above the thresholds of ECAPs and subsequently in time (Fig. 2), the intraspinal gray matter sources correlated with ESAPs (Fig. 4), and the sensitivity of ESAPs to the AMPAR antagonist CNQX (Fig. 3), support the hypothesis that ESAPs reflect the synaptic excitatory inputs evoked by SCS. We consider potential origins of ESAPs as they may relate to the mechanisms of action of SCS or, more broadly, measure the “state” of the spinal cord.

ESAPs may reflect the excitatory inputs from large diameter sensory afferents (e.g., dorsal column fibers) to dorsal horn neurons. Features of the S1 ESAP are consistent with intracellularly recorded monosynaptic responses of dorsal horn neurons to dorsal column stimulation from in vitro models (Baba et al., 1994). ESAPs may thus suggest processing of sensory input (e.g., Aβ afferents) in the dorsal horn (Abraira and Ginty, 2013). Dorsal horn neurons with dendrites close and articulated toward the epidural recording electrodes could be sources for the ESAP, consistent with the intraspinal S1-wave recording (Fig. 4*B*). Furthermore, some dorsal horn neurons that receive large diameter inputs ramify dorsoventrally across the superficial and deep dorsal horn (Woolf and King, 1987), potentially explaining why substantial responses were observed in both superficial and deep dorsal horn with intraspinal electrodes. The correspondence between signals evoked by both dorsal column and cortical stimulation (Fig. 4*C*), at a minimum confirm the detectability of synaptic activity originating in spinal circuits and may further suggest a convergent neuronal source possessing glutamatergic synapses from dorsal column collaterals and descending cortical projections (Abraira et al., 2017).

Under the conditions tested, the ESAP (but not ECAP) exhibited gradual adaptation following 50-Hz stimulation (Fig. 7). Habituation is consistent with (though not exclusive to) synaptic processes (Shimoji et al., 1982; Sdrulla et al., 2015) and is also shown for superficial dorsal horn interneuron responses to 50-Hz SCS (Fan et al., 2022).

Alternatively, the ESAP signal may reflect activation of descending fibers (e.g., via current spread to the dorsolateral funiculus) that synapse onto the dorsal horn and, more broadly, descending modulation (distinct from antidromic segmental effects) by SCS (Saadé et al., 2015). This conjecture is consistent with the above-ECAP threshold intensities to evoke ESAPs, correspondence to cortical evoked responses, and suppression by CNQX.

All these hypotheses indicate ESAPs represent an epidurally measurable but heretofore uncharacterized indicator of dorsal horn (e.g., sensory or nociceptive) processing. Notwithstanding the motivation for further studies regarding the neural origins of the ESAP signal and translational applicability, our SCS experimental series in the context of decades of background studies on spinal electrophysiology show ESAPs may be rich sources of information on spinal cord state.

Also, ESAPs may have broader implications beyond a marker of sensory modulation. Intraspinal synaptic activity, evoked by either spinal stimulation or cortical stimulation (corticospinal tract; CST), can be detected by epidural electrodes. Where cortical or spinal electrical stimulation is used for spinal plasticity-induction (as the case for neuro-rehabilitation following injury), ESAPs can be used to provide an indicator of both acute synaptic modulation (e.g., spike timing-dependent plasticity, STDP) and lasting changes in spinal motor circuits.

### Limitations

The translational applicability of ESAPs to clinical therapies requires further studies. For example, studies need to be performed to assess whether ESAPs are directly correlated with therapy and/or disease-state, as this association will elucidate their applications to neuromodulation.

Experiments were performed on anesthetized but otherwise healthy animals, so conclusions regarding ESAPs cannot be simply extended to chronic and/or neuropathic pain states. However, this limitation does not detract from the possibility that ESAPs reflect network activity in the dorsal horn gray matter and motivate future work with injury and disease models. Absolute currents thresholds will also vary across conditions (animal model, anesthesia, stimulation frequency/waveform/montage, surgical procedures, etc.). Despite the absolute variability, ECAP generally had lower thresholds than ESAP (Fig. 5) and EMG thresholds are approximately ∼2× above even S1, and our reported EMG thresholds being 3× above ECAPs are consistent (or conservative) compared with prior studies (Cedeño et al., 2022; Dietz et al., 2022).

CNQX is pharmacologically selective to AMPA receptors, but cannot distinguish specific cell or fiber input types, however intrathecal CNQX delivery supports segmental rather than systemic actions, as do intraspinal recordings of the dorsal horn. It is possible that unrecorded muscle EMG contaminated the signal, but this would be inconsistent with the nature of S1 responses (e.g., inversion of polarity across segmented and within the spinal cord) and another rodent study using a similar stimulation and recording geometry confirm epidural recordings were unaffected by (local) EMG responses (Cedeño et al., 2022). We characterized one ESAP, the S1 wave, and additional ESAP signals should be considered, that may involve distinct neurotransmitters or polysynaptic connections. Indeed, our demonstration that ESAPs can be detected during SCS motivate and methodologically inform further mechanistic and application studies.

## Synthesis

Reviewing Editor: Jeffery Twiss, University of South Carolina Decisions are customarily a result of the Reviewing Editor and the peer reviewers coming together and discussing their recommendations until a consensus is reached. When revisions are invited, a fact-based synthesis statement explaining their decision and outlining what is needed to prepare a revision will be listed below. The following reviewer(s) agreed to reveal their identity: David Magnuson.

This revised manucript has been seen by the original reviewers. Both noted that it is improved and felt that it will provide an advance for the field. They noted that you had been responsive to the critiques and largely addressed the concerns. In particularly, one reviewer noted that the increase of clinical applications for epidural stimulation emphasize a need for a greater understanding of the neurophysiological mechanisms responsible for the recovery, and some of the answers could in part lie in the S1 responses discovered by the authors. However, a reviewer also noted that some of the additions raise issues and, from our online discussion, we think these need to be addressed before further considering for publication.

Major Issues:

The discussion is now longer than is necessary, is somewhat redundant given the list of “evidence” and the following text. Please streamline your discussion to focus on the most important, major points that are raised by your data and temper your speculations (see below).

Though the initial submission critiques did suggest for you to speculate on the nature of the S1 responses, the reviewers ask that you soften that speculation some. The discussion of the superficial dorsal horn circuitry does not seem to be in keeping with the results that show substantial response amplitude from the deeper dorsal horn laminae. The concept that the responses may involve nociceptive responses was not considered to be supported (or at least not explicitly supported) by the data.

Minor Issues:

Line 20: Please define ESAP before using the acronym in the abstract.

Line 83. Should probably indicate that “future studies on”, rather than ongoing, even if they are ongoing.

Lines 194/5. This is a critical statement referring to the stimulation parameters used, and the term “dose” may be somewhat loose for this. I suggest being more concise, perhaps stating to “normalize the stimulation parameters” or to “normalize the stimulus amplitude”. Later, on lines 199/200, please clarify what the “specific visible movement of the hindlimb” entailed. Was it an extension of one or more joints, or something else?

Lines 255/6. “For back EMG experiments” is confusing. I assume you mean the LT EMG. Please use thoracic, trunk or axial EMG to describe this. It comes up again on line 420. This also relates to line 244, where you first describe the recording. Please use trunk instead of back.

Lines 285/7. This should probably state that the lidocaine was administered and the particulars of the stimulation/recordings that were then repeated.

## Author Response

Synthesis of Reviews:

Computational Neuroscience Model Code Accessibility Comments for Author (Required): N/A

This manuscript has been assessed by two reviewers and the reviewer editorial board member. The two reviewers were initially of mixed decisions on this submission, but they came to a consensus in the online discussion. In situations like this, I typically append the individual reviewers’ comments to this synthesis statement, and focus my comments to the online discussion. Both reviewers agreed that the experiments are rather elegant and very well done, and the data are convincing. Reviewer 2 pointed out their main concern is the lack of insights into what those S1 responses may do or represent, which makes the study purely descriptive.

Reviewer 1 raised the point that going beyond speculations would require solid evidence for what the stimulation does - both agreed that developing experiments that would establish the function of the S responses will be a major undertaking, but would allow this work to go beyond merely descriptive. Both felt that logical speculation of what the ESAPs mean and what circuitry they might involve could be sufficient to consider resubmission. Reviewer 1 also noted that relationship with EMG latencies/amplitudes does bring some interesting things to mind.

We appreciate the feedback that the experiments are ‘elegant’ and ‘well done’. The hypothesis supported by these experiments is that the S1 is a signal reflecting grey matter synaptic activity detectable with epidural electrodes- distinct from ECAPS which reflect only white matter (non-synaptic) activity. In the revision we are more explicit why this is a potentially unique and high value signal more directly related to spinal gray matter processing during SCS. Given SCS therapy is presumed to derived from grey matter synaptic activity, this is a straightforward argument that is indeed (following this paper) testable in future studies - as we have elucidated the source of the S1 signal in this paper. The impact of our paper’s discovery may therefore be broader than the impact that ECAPs have made with respect to understanding SCS mechanisms. As indicated the Discussion expands on the meaning of ESAPs to relevant circuitry.

Reviewer 1:

This manuscript describes a really quite elegant series of experiments investigating the spinal response to epidural spinal stimulation using what I believe are clinically relevant approaches in the adult rat model. It includes recordings made from an epidural electrode, identical to the spinal cord stimulating electrode, recordings made from an intraspinally placed extracellular electrode array capable of recording from intermediate gray matter, and finally recordings made of EMG from the hindlimb TA muscle. Stimulations were delivered via the epidural stimulating electrode mentioned above, and in addition via an electrode placed over the hindlimb motor cortex. The investigators use CNQX to evaluate the component of the evoked signal they are referring to as ESAPs (evoked synaptic activity potentials), and the results are very convincing.

We thank Reviewer 1 for the encouraging comment on paper ‘elegance’ and ‘clinically relevant’.

Reviewer 1’s constructive criticism is systematically addressed below.

2

Some questions that I think should be better addressed in the paper are the following. 1. Why choose T12 to stimulate and L1 to record? Please provide a stronger rationale/explanation for this. 2. Please clearly explain the relationship between the bone and spinal cord with respect to electrode positions. There is some confusion and the figures just make this confusion worse. Is each laminectomy a multi-level laminectomy (T10 and T11, plus T13, L1 and L2)?

1. As conventional in rodent studies, the stimulation electrode was adjusted to selectively and optimally activate the hindlimb (EMG). This allows relation of stimulation amplitude with EMG threshold (this is generally used in SCS rodent research, though in our case was also used to rule out myogenic contribution to ESAPs). Vertebral locations were chosen based on prior work demonstrating that vertebral levels T12-L1 correspond to lumbar spinal dermatomes (e.g., L2-L5) often explored in chronic pain and motor systems research (Gelderd and Chopin, 1977; Gerasimenko et al., 2019; Shechter et al., 2013; Swett and Woolf, 1985; Tao et al., 2021). Lumbar region houses the motorneurons that innervate the muscles of the lower extremities. This is also a region that is commonly used in the studies for pain management and also in cases of rehabilitation. We wanted the study design (stimulation and recording) to be relevant for the further exploration of relevance of our results in pain relief by SCS. As a result, we chose L2-L5 spinal segment as our recording site.

2. The methods now clarify the laminectomy procedure. Yes, each laminectomy is a multi-level laminectomy. A new section has been added to the methods - ‘placement of epidural leads’ that clarifies the relationship between the bone and spinal cord with respect to electrode positions. Overall, the evidence that the ESAP is, in fact, a multi-synaptic response involving interneurons located fairly deeply within the dorsal horn or intermediate gray matter is quite convincing and it was somewhat refreshing to have the nitty gritty rule-in, rule-out arguments laid out so nicely. The time-frame of wash-in and wash-out, for example, is pretty key to the argument and these components are well done and well explained. We appreciate the reviewer’s acknowledgment of the pains we took to generate multiple lines of converging evidence. In the paper revision, still more animals (n) are included as well as recordings from additional muscle groups and a lidocaine control, which further bolster our conclusions.

Significance statement: It would be logical to also include the use of SCS/epidural stimulation for spinal cord injury.

We agree on this further significance for our paper, now noted.

Also in the significance statement is a typo ... it should be immediately not immediacy.

Updated to “direct”.

Abstract: A nit-picky detail, but on line 11 the authors refer to recording from epidural and intraspinal electrodes, but they also record from the EMG electrode, correct? Yes. Clarified. 3

Also in the abstract, on line 14 they state that the S-1 wave has a distinct dose -response and I think that should be clarified to be a stimulus-intensity dose-response (or however they want to put it) ... if they are referring to the CNQX dose response, this needs to be mentioned here, not in the next sentence.

Clarified.

Intro: In the concluding paragraph of the intro you state “ESAPs...provide separate information on spinal cord state.” Yes, this is true but not very satisfying. Please qualify the “state”, and what you mean by that. I think they have underplayed the importance of the work. Edited. We appreciate the encouragement to clarify the importance of the work. Surgery and electrode placement. The surgery description implies a bilateral laminectomy at T10-T11 (2 levels?) and a second “on the left side” of the T13-L2 vertebrae. Is this accurate and could more detail be provided please? This goes along with #2 above.

Method section has been updated to clarify the laminectomy procedure and the placement of epidural leads.

Also, on page 9 the authors talk about the spinal stimulation electrode being repositioned...is this equivalent to the mapping that many clinical folks report for epidural stimulation? Was this repositioning done mid-experiment or only at the beginning? Why was it necessary? What about the new position allowed it to access the circuitry with a low movement threshold. This seems kind of fuzzy and confusing?

We clarified these methodological details. The repositioning was done before starting the experimental recordings to ensure that the sites used to stimulate could elicit hindlimb movement at a low stimulation threshold and S1- wave recorded using a different epidural lead. See point above on EMG isolation. The final paragraph on page 25 is very important and I think is somewhat obtuse in its conclusion(s). The first sentence is confusing and that leads to further confusion later in the paragraph. Does this relate to the studies where cortex stimulation is paired with other stimuli and/or voluntary efforts to increase signals below an injury (in the clinical population)?

The paragraph has been fully re-clarified. We agree there is a further important impact of this work but to also avoid speculating too far.

Reviewer 2:

The manuscript under evaluation presents an analysis of a delayed epidural/intraspinal response called “S1” produced by epidural stimulation that is based on synaptic activity within the spinal cord. The manuscript presents recordings of the S1 responses in the caudal cord epidural electrode following bipolar stimulation at more rostral sites (Fig. 2) and also recordings of the effects of CNQX (a AMPA antagonist) on those responses (Fig. 3). The paper also demonstrates that the S1 responses can be observed on intraspinal recordings in the dorsal to intermediate zone of the grey matter in response to epidural or cortical stimulation and that those responses are also sensitive to CNQX administration while shorter latencies responses (the traditional P1,N1,P2 & N2) are not suppressed. The manuscript presents evidence that S1 4 responses predate EMG responses, and are thus not an artifact of the EMGs (the rather long latencies (6ms or so) between S1 and EMGs also suggest that S1 is not a premotor response). Overall, the evidence presented is rather compelling that the S1 responses are synaptically mediated, and not artifacts.

We thank Reviewer 2 for the encouraging feedback and confirmation our experimental designs addressed our main goal. Reviewer 2’s valued critiques are systematically addressed below. Some of the current values used for cortical and epidural stimulation seem relatively large compared to other groups and that raises some questions about the neural elements activated. Date presented for cortical stimulation for example used currents up to 4mA, while the M1 mapping experiments cited by the authors that used a similar stimulating electrode used maximum currents of 1.5mA (Song and Martin, 2017). The value for epidural stimulation are also on the high side compared with certain groups, but in line with others. Song and Martin used maximum currents of 1.5 mA that was also the threshold for visual motor response evoked by short stimuli train of 13 pulses at 333 Hz in their study (Song and Martin, 2017). The visual motor thresholds (vMTs) for cortical stimulation in our studies align with their studies (>2 mA). The vMTs were determined using the similar stimuli used in their study (train of 13 pulses at 333 Hz). The threshold current (2 mA) when applied to M1 HLA elicited a distinct epidural spinal response (Fig. 4C1). The higher current amplitude (4 mA) was chosen to be able to clearly discern the effect of CNQX on the epidural spinal response.

As suggested by the reviewer, for epidural spinal stimulation, our current values obtained using a 40 μs pulse width align with the studies using shorter pulse widths (Dietz et al., 2022; Parker et al., 2013). Neurons have higher thresholds for excitation as the stimulus pulse width decreases (Gmel et al., 2021; Molnar and Barolat, 2014), in accordance with standard strength-duration relationships. The manuscript is well written and relatively easy to read, although the abstract could benefit from a reduction in the number of acronyms used. Figures are of high quality and present the information clearly. Thank you, we endeavored for clarity. Besides the concerns about stimulation levels, the main weakness of the manuscript is that the authors do not address the potential functional role of the S1 responses. Based on their relative threshold of activation and relative latencies to stimulation and motor responses, the manuscript should be presenting some hypotheses about their roles in either sensory processing or motor output. The manuscript appears to be an attempt to claim the discovery of a new phenomenon, but does little to explain what the function/role/importance of the phenomenon is. An expansion of the discussion about the potential roles, and whether those hypothesized roles are supported by the neurophysiological evidence presented would greatly increase the significance of the results.

Minor comments:

Lines 195-196: “lowest current amplitude (100μA) as commonly used in M1 mapping experiments (Song and Martin, 2017; Takemi et al., 2017)”. Song and Martin report thresholds in mA (except for intracortical where thresholds drop to μA) and Takemi et al. report using levels 5 in the 0.5mA range. The authors may want to consider dropping the 100μA range reference which seems very low for surface cortical stimulation, especially compared with the 4mA they used.

Corrected.

Line 494: “post-stimulation pule time”, change to “pulse”.

Corrected.
