# Supplementary figures and images for: Novel Evoked Synaptic Activity Potentials (ESAPs) Elicited by Spinal Cord Stimulation
Source: eNeuro. 2023 May 16;10(5):ENEURO.0429-22.2023. doi: 10.1523/ENEURO.0429-22.2023 (PMC10198607; doi:10.1523/ENEURO.0429-22.2023)

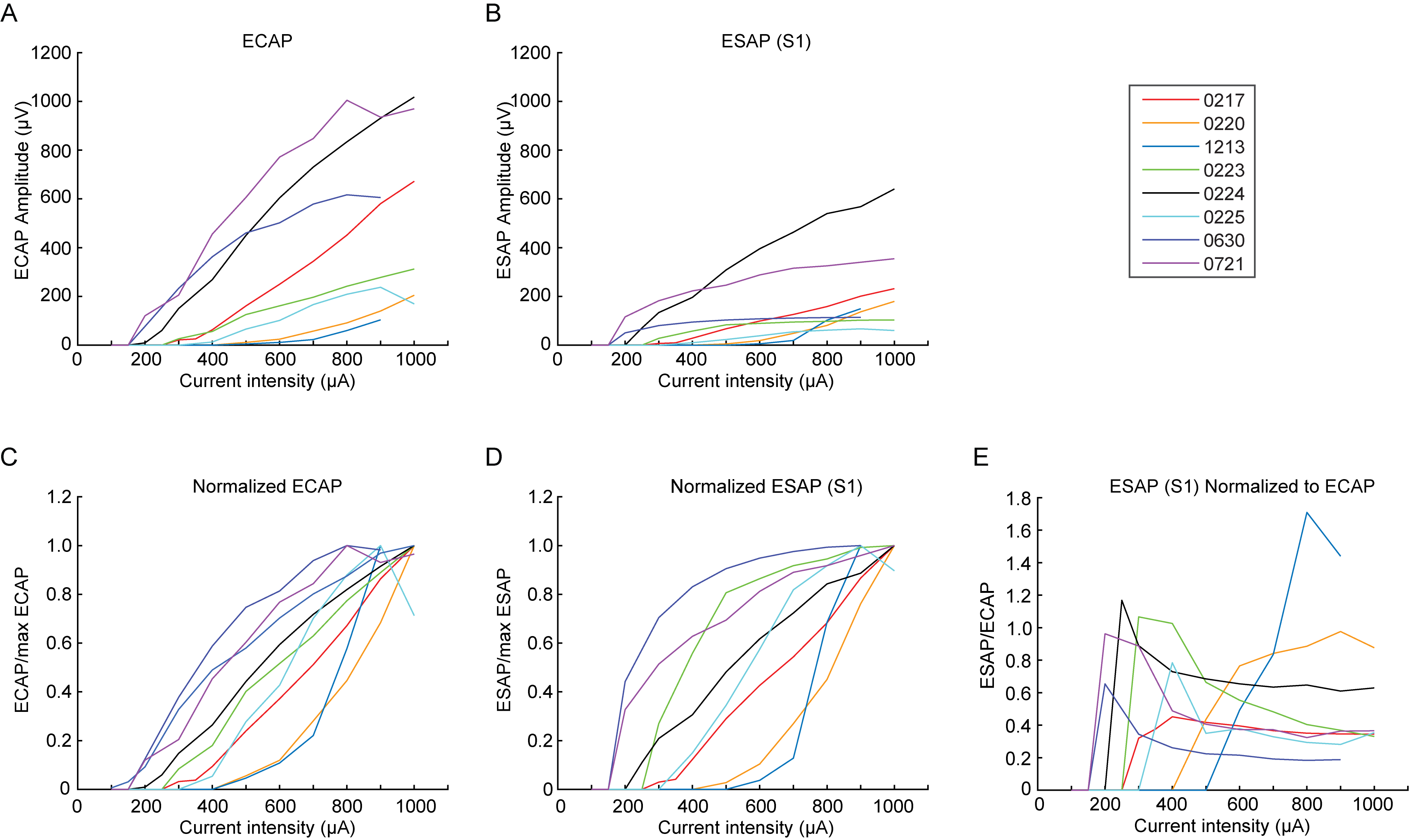

Supplement: Extended Data Figure 2-1 — Dose–response curve for ESAP (S1) and ECAP. Monotonic increase in the amplitudes of ECAP (A) and ESAP (S1; B) with increasing stimulation intensity across eight animals. C, D, Both ECAP and ESAP (S1), when normalized to the maximum evoked response, exhibit a monotonic progression with increasing stimulation intensity. E, The evoked ESAP (S1), when normalized to corresponding evoked ECAP, show a nonmonotonic progression with increasing current. Data represented as mean of 20 trials from eight animals (color coded). Figure Contributions: Mahima Sharma and Vividha Bhaskar performed the experiments. Mahima Sharma analyzed the data. Download Figure 2-1, TIF file. [file enu-eN-NWR-0429-22-s02.tif]
